# Efficacy of Unregulated Minimum Risk Products to Kill and Repel Ticks

**DOI:** 10.3201/eid3001.230813

**Published:** 2024-01

**Authors:** Lars Eisen

**Affiliations:** Centers for Disease Control and Prevention, Fort Collins, Colorado, USA

**Keywords:** ticks, efficacy, toxicants, repellents, minimum risk products, unregulated, vector-borne infections, zoonoses, United States

## Abstract

Human-biting ticks threaten public health in the United States. Registration by the Environmental Protection Agency of products to kill host-seeking ticks or repel ticks contacting humans is indicative of their safety and effectiveness. Unregulated minimum risk products, exempt from Environmental Protection Agency registration and often based on botanical oils, are proliferating in the marketplace, but there is concern about their effectiveness to kill and repel ticks. Evaluations of such products are limited in the published literature. A review showed considerable variability among minimum risk products to kill host-seeking blacklegged ticks, with effectiveness similar to chemical pesticide products for some minimum risk products but minimal impact on the ticks for other products. Evaluations of minimum risk tick repellents have typically focused on individual active ingredients rather than formulated products, which often combine multiple active ingredients. Consumers should be aware that effectiveness to kill and repel ticks can differ among unregulated minimum risk products.

Human-biting ticks threaten public health in the United States. The blacklegged tick (*Ixodes scapularis*) is a frequent human-biter and vector of viral, bacterial, and parasitic agents causing human illness, including Lyme disease (*1*). Strategies to prevent human tick bites include broadcast of pesticide products (toxicants) to kill host-seeking ticks in the environment and potentially reduce the likelihood of encountering ticks, as well as use of repellent products applied to skin or clothing to reduce the chance of a tick encounter leading to a bite (*2*). In both cases, consumers can choose to use either products registered by the US Environmental Protection Agency (EPA) or minimum risk products, also called 25(b) exempt products, that are exempt from EPA registration because the active and inert ingredients they contain are considered to pose little to no risk to human health or the environment (*3*).

Active ingredients allowable in a minimum risk 25(b) exempt product include botanicals (e.g., cinnamon, citronella, cloves, garlic, peppermint, rosemary, sesame, spearmint, thyme, and white pepper), botanical oils (e.g., castor oil, cedarwood oil, cinnamon oil, citronella oil, clove oil, corn oil, eugenol oil, garlic oil, geraniol oil, geranium oil, lemongrass oil, linseed oil, peppermint oil, rosemary oil, sesame oil, soybean oil, spearmint oil, and thyme oil), and some other types of compounds (e.g., citric acid, lauryl sulfate, malic acid, potassium sorbate, and sodium chloride) (*4*). Most compounds allowable as active ingredients are readily understood by the public to be of natural origin. Active ingredients in EPA-registered tick toxicant or repellent products often represent synthetic compounds (e.g., N,N-diethyl-meta-toluamide [DEET], IR3535, and picaridin for skin repellents; permethrin for clothing treatment; and various carbamates and pyrethroids to kill host-seeking ticks) but can also be compounds of natural origin in the case of repellents (citronella, citronella oil, and oil of lemon eucalyptus) (*5*).

Consequently, antitick products on the market fall into 3 categories: unregulated products based on minimum risk 25(b) exempt active and inert ingredients; EPA-regulated products containing active ingredients of natural origin; and EPA-regulated products containing synthetic (chemical) active ingredients. Citronella is unique in that it is included both in some EPA-registered repellent products labeled for ticks (*5*) and in minimum risk 25(b) exempt tick repellent products.

Registration by EPA of products to kill host-seeking ticks or repel ticks contacting humans is indicative of product effectiveness. Unregulated minimum risk 25(b) exempt products are proliferating in the marketplace but there is concern about their effectiveness to kill and repel ticks, as expressed in the 2020 report by the Tick Biology, Ecology, and Control subcommittee of the Tick-Borne Disease Working Group established by the US Department of Health and Human Services (*6*). In this perspective, I focus on what is known about the efficacy of EPA-registered versus minimum risk 25(b) exempt products to kill and repel ticks, and how end-users choose among these product types.

## Consumer Choice of EPA-Registered versus Unregulated Minimum Risk 25(b) Exempt Tick Toxicant and Repellent Products

Surveys conducted in Lyme disease‒endemic areas of the United States provide information on levels of use of synthetic versus natural tick toxicants (*7**–**9*) and repellents (*8*,*10*). However, survey questions were not phrased specifically to distinguish between EPA-registered and minimum risk 25(b) exempt products. Moreover, no survey has addressed the reasons why members of the public choose to use EPA-registered versus minimum risk 25(b) exempt tick toxicant and repellent products. A reasonable assumption is that choice of an EPA-registered product is driven by a belief that the registration ensures the product will be safe and effective in killing or repelling ticks when used according to label recommendations. Use of EPA-registered products also is recommended by public health agencies, including the Centers for Disease Control and Prevention (*11*). Conversely, it is reasonable to assume that choice of a minimum risk 25(b) exempt product is driven, in part, by the belief that it is safer than a synthetic product for the user, other family members, pets, wildlife, or the environment. It is not clear to what extent the consumer assumes a minimum risk 25(b) exempt product will be as effective as an alternative EPA-registered product in killing or repelling ticks.

### Tick Toxicants for Use in the Environment

Two studies in the northeastern United States (*7**,**8*) each reported similar levels of use of synthetic versus natural products to kill host-seeking ticks on residential properties. One study (*7*) used the phrasings chemical pesticide (reportedly used by 23% of respondents to control ticks) and natural pesticide (reportedly used by 15% of respondents). However, it is not clear if pesticides considered to be natural by the respondents were minimum risk 25(b) exempt products. The other study (*8*) used the phrasings of synthetic pesticides (e.g., bifenthrin) versus natural/organic pesticides (e.g., cedar oil), with both types of pesticides reportedly being used by 2%–3% of respondents to control ticks. Those phrasings provided an example of an EPA-registered active ingredient (bifenthrin) and a minimum risk 25(b) exempt active ingredient (cedarwood oil) but still fall short of explicitly comparing EPA-registered versus minimum risk 25(b) exempt products.

Another study explored willingness to pay for natural versus chemical pesticide yard treatments to kill host-seeking ticks (*9*). Of those willing to pay for yard treatment, 95% were willing to use a natural pesticide, compared with 63% for a chemical pesticide. In both cases, most respondents (63%–66%) were only willing to pay up to $99/year for the treatment, and few respondents (<5%) were willing to pay $500 or more. Additional information came from an assessment of commercial tick control practices on residential properties in the Northeast (*12*). Most (80%) firms offering tick control services reported applying synthetic pesticide products (carbaryl and various pyrethroids) to kill host-seeking ticks. A smaller proportion (34%) of firms reported applying natural or organic pesticide products; those were based on a variety of natural active ingredients, including minimum risk 25(b) exempt compounds (cedar oil, geraniol oil, peppermint oil, rosemary oil, and thyme oil) and other compounds of natural origin (pyrethrin). Primary reasons for the firms to not offer natural products to kill host-seeking ticks included efficacy concerns, followed by lack of client request and cost. In an earlier survey in the northeastern United States, safety concerns was the most common reason for not using synthetic pesticides for tick control on residential properties, but similar information was not provided for natural pesticides (*13*).

### Tick Repellents

Two studies in the Upper Midwest (*10*) and northeastern (*8*) United States each reported similar levels of use, or willingness to use, for synthetic versus natural tick repellents. The survey in the Upper Midwest made the distinction between natural and synthetic repellents (respondent preference of use among these product types was 24% for natural repellents only versus 19% for synthetic repellents only), but the EPA-registered oil of lemon eucalyptus active ingredient was used as the example of a natural repellent (*10*). Therefore, the survey findings cannot be interpreted in the context of EPA-registered versus minimum risk 25(b) exempt repellents. The survey in the Northeast (*8*) used the more explicit phrasings of spray containing EPA-approved repellent (e.g., DEET), which reportedly was being used by 13%–17% of respondents, versus natural/organic spray repellent, which reportedly was being used by 8% of respondents. However, it is not clear in which category respondents would place an EPA-approved repellent based on compounds understood to be of natural origin, such as oil of lemon eucalyptus or citronella.

## Efficacy of Unregulated Minimum Risk 25(b) Exempt Products to Kill Host-Seeking Ticks

With 1 notable exception (*14*), field trials of commercial minimum risk 25(b) exempt products have compared a single product with a negative control (untreated or sprayed with water) and often also a positive control (an EPA-registered synthetic pesticide). Additional factors to consider when interpreting the results of such field evaluations include whether sprays were applied at low or high pressure and the timepoints sampled after application. Products based on synthetic pyrethroids effectively suppress host-seeking blacklegged ticks for at least 6 weeks, with similar results for low- and high-pressure spray applications (*15*). Those pesticides are stable in the environment and their efficacy is not dependent on being applied at high pressure to increase penetration of vegetation and the litter and duff layers; they will affect both the ticks they reach during the spray event itself and ticks that contact them weeks later while moving around in duff and litter layers or ascending vegetation while seeking a host. Minimum risk 25(b) exempt pesticide products appear to be less stable in the environment and therefore highly effective in suppressing blacklegged ticks only for a shorter period of time, often 1–3 weeks, thus requiring more frequent applications to achieve the same level of tick suppression as synthetic pesticides (*15*). It may be that efficacy of minimum risk 25(b) exempt pesticide products to suppress host-seeking ticks is higher when they are applied at high spray pressure and therefore are able to penetrate vegetation and the litter and duff layers to reach ticks more effectively during the application event. Application of EPA-registered synthetic pesticide products labeled for ticks has, with 1 notable exception (*16*), uniformly resulted in high (>80%) tick killing efficacy (*15**,**17**–**20*). In contrast, tremendous variability in killing efficacy has been observed among different minimum risk 25(b) exempt pesticide products, including for a study using standardized methods to simultaneously compare multiple minimum risk 25(b) exempt pesticide products (*14*).

### Products Based on Rosemary and Peppermint Oils

Multiple studies have investigated the efficacy of minimum risk 25(b) exempt products containing rosemary and peppermint oils to suppress blacklegged ticks when applied to naturally infested field plots. Two studies conducted in Maine (*21**,**22*) evaluated the product Eco-Exempt IC2 (containing 10% rosemary oil and 2% peppermint oil). Applied by high-pressure spraying by a pest control firm on a single occasion, this minimum risk 25(b) exempt product was as effective as a positive control product (SpeckoZ) containing the synthetic pyrethroid bifenthrin in reducing the abundance of host-seeking blacklegged ticks for several months after application. Two other studies conducted in New Jersey (*17**,**23*) evaluated similar products: EcoTrol T&O (containing 10% rosemary oil, 2% peppermint oil, and 0.5% sodium lauryl sulfate) and Essentria IC^3^ (containing 10% rosemary oil, 5% geraniol oil, and 2% peppermint oil). Applied by low-pressure spraying, those products did not maintain a high level (>90%) of suppression of nymphal blacklegged ticks for more than 1–3 weeks and required multiple applications to remain moderately to highly effective (>70% suppression) over a longer period. In the study with Essentria IC^3^ (*17*), a positive control product (Talstar P) containing bifenthrin provided 100% suppression of nymphal blacklegged ticks for 9 weeks after a single spray event. A follow-up study (*24*) to compare the effect of Essentria IC^3^ applied by low-pressure versus high-pressure spraying did not find an extended duration of suppression for nymphal blacklegged ticks with the high pressure-spraying: regardless of spray pressure, the level of suppression decreased to <60% after 2 weeks and 20% after 3 weeks. Moreover, the low-pressure spraying unexpectedly outperformed the high-pressure spraying to suppress nymphs at some timepoints after application in this trial.

Both Eco-Exempt IC2 and Essentria IC^3^ also were evaluated in a standardized field microplot trial where nymphal blacklegged ticks were introduced into field arenas (*14*). Eco-Exempt IC2 showed 87% suppression of ticks placed in the arenas before spraying (knockdown effect), but when ticks instead were introduced 2 weeks after the spray event (residual effect), the level of suppression fell to 30%. Essentria IC^3^ was evaluated in 3 different years in the study; and knockdown suppression ranged from 15% to 53% and residual suppression from 0% to 6%. Two additional products (Private Label 1 and 2), based on the original Eco-Exempt IC2 formulation and including rosemary oil, peppermint oil, and geraniol oil, also were evaluated: knockdown suppression ranged from 0% to 37% and residual suppression from 0% to 17%. A follow-up study (T.N. Mather, University of Rhode Island, pers. comm., 2023 Aug 16) using the same experimental system reported low levels of knockdown suppression (0%–16%) and residual suppression (0%–15%) for multiple products, based on from rosemary or peppermint, together with oils from clove and thyme. Knockdown and residual killing efficacy for Talstar P (bifenthrin) were 98%–100% in both studies (*14*; T.N. Mather, University of Rhode Island, pers. comm., 2023 Aug 16). The highly variable findings across different studies for minimum risk 25(b) exempt products based on rosemary and peppermint oils underscore the difficulty in making recommendations about unregulated products based solely on the active ingredients they contain.

### Products Based on Cedarwood Oil

Natural product pesticides based on cedarwood oil are commonly offered by commercial firms providing tick control services in the northeastern United States (*12*). Laboratory studies (*25**–**27*) have demonstrated toxicity of cedarwood oil toward multiple tick species, including the blacklegged tick, but there are no published data on the efficacy of minimum risk 25(b) exempt products based on cedarwood oil to suppress blacklegged ticks in naturally infested areas. However, 2 products based on cedarwood oil (CedarCide PCO Choice and Tick Killz) were evaluated in the standardized field microplot system (*14*). Neither product provided more than minimal tick knockdown (5%–6%) or residual tick suppression after 2 weeks (0%–8%). A follow-up study (T.N. Mather, University of Rhode Island, pers. comm., 2023 Aug 16) using the same experimental system found similar results for 3 products based primarily on cedarwood oil; tick knockdown ranged from 0% to 24% and residual tick suppression from 0% to 15%.

### Product Based on Garlic

A product called Mosquito Barrier (99.3% garlic juice, 0.5% citric acid, and 0.2% potassium sorbate), labeled as repellent rather than toxicant, was evaluated on naturally tick-infested plots in Connecticut (*28*). A laboratory trial showed the product to repel but not be toxic to blacklegged ticks at the label application rate (*28*). When applied by high-pressure spraying in the field, the product provided short-term (1–3 weeks), moderate (37%–59%) suppression of host-seeking nymphal blacklegged ticks, presumably due to repellency keeping the nymphs down in grass thatch or leaf litter rather than a toxicant effect.

## Efficacy of Unregulated Minimum Risk 25(b) Exempt Products to Repel Ticks

Studies on repellency against ticks of natural substances, or fractionated compounds from these substances, were reviewed previously (*29**–**32*). Experimental studies typically focused on active ingredients; published studies comparing commercial products available in the United States are rare and restricted to EPA-registered products (*33**–**36*). EPA registration indicates a product will effectively repel ticks for a label-specified duration of time, and the online tool for repellent products provided by the EPA (*5*) indicates the number of hours protection is expected to last for a specific product. Essential considerations (already accounted for in the case of EPA-registered repellents) regarding minimum risk 25(b) exempt repellent products include their efficacy to repel ticks, as well as the duration of repellency after application. Similar to tick toxicant products (*6*), there is concern that minimum risk 25(b) exempt tick repellent products might be less effective and have a shorter duration of protection than repellent products based on synthetic chemicals, such as DEET or picaridin. The evaluations outlined here focus specifically on minimum risk 25(b) active ingredients and repellent products, excluding studies on fractionated compounds from the active ingredients.

### Minimum Risk 25(b) Exempt Commercial Repellent Products Available in the United States

Published evaluations of minimum risk 25(b) exempt repellent products are limited to 3 studies (*37**–**39*). Two studies (*37*,*38*) focused on EcoSMART Organic Insect Repellent (containing 1% geraniol oil, and 0.5% oils of each of rosemary, cinnamon, and lemongrass). The repellency of this product was compared with a permethrin product (Repel Permanone) by application to tick drags that then were used to collect ticks from natural areas (*37*) or to coveralls used by the investigators to walk through tick-infested vegetation (*38*). The repellent efficacy (based on numbers of ticks still remaining on treated versus untreated textile 3 min after contact with vegetation ended) of the EcoSMART Organic Insect Repellent was similar to that of Repel Permanone for blacklegged ticks and lone star ticks (*Amblyomma americanum*). Repellency was uniformly >90% against both tick species up to 2 days after textiles were treated with the EcoSMART Organic Insect Repellent. The third study (*39*) was on experimental formulations called TT-4228 and TT-4302 (containing 5% geraniol oil as the active ingredient), subsequently marketed under the product name Guardian. Those experimental formulations were as effective as a 15% DEET product (OFF! Active Insect Repellent) in repelling blacklegged ticks, lone star ticks, American dog ticks (*Dermacentor variabilis*), and brown dog ticks (*Rhipicephalus sanguineus* sensu lato) in an in vitro assay, and TT-4228 outperformed the DEET product in repelling lone star ticks when applied to socks worn in a field trial (treatments applied 2.5–3.5 hours before the trial). Although the results of the field trials with minimum risk 25(b) exempt repellent products outlined above are promising, none included evaluation of application to skin, which might differ in repellency from application to textiles. Published data from laboratory studies using the EPA-recommended human skin bioassay (*40*) to assess repellency are entirely lacking for minimum risk 25(b) exempt repellent products labeled for use against ticks. To be most informative, such studies should include the main human-biting life stages of multiple tick species of medical concern.

### Evaluations of Minimum Risk 25(b) Exempt Active Ingredients against Ticks of Medical Concern in the United States

A recent study (*41*) compared the repellency of 19 minimum risk 25(b) exempt active ingredients (as 10% lotion emulsions) against female blacklegged ticks by using the EPA-recommended human skin bioassay. Complete protection times in this assay ranged from less than 10 minutes (castor oil, corn oil, cottonseed oil, linseed oil, rosemary oil, sesame oil, and soybean oil) to more than 10 minutes but less than 1 hour (cedarwood oil, citronella oil, cornmint oil, garlic oil, geranium oil, lemongrass oil, peppermint oil, spearmint oil, and thyme oil) and 1‒2 hours (cinnamon oil, clove oil, and geraniol oil). No minimum risk 25(b) exempt active ingredient had a complete protection time >2 hours, whereas the positive DEET control provided complete protection for the entire 6-hour observation period. Similar results for peppermint oil and rosemary oil against nymphs of the blacklegged tick were reported for human skin bioassays in another recent study (*42*): a positive DEET control remained effective (>80% repellency) over a 6-hour period, whereas initially high repellent efficacy of peppermint oil fell below 20% after 2 hours, and rosemary oil was not repellent at any timepoint after application. Another study (*43*) compared repellency of multiple minimum risk 25(b) exempt active ingredients against nymphal lone star ticks in an in vitro assay: lower concentrations of clove oil and thyme oil repelled 95% of ticks, compared with cinnamon oil, cedarwood oil, and peppermint oil. Additional studies, using variable methods to assess repellency for 1 or 2 minimum risk 25(b) exempt active ingredients, included evaluations of repellency against blacklegged ticks or lone star ticks for cedarwood oil (*27*,*44*), geraniol oil (*45*), geranium oil (*46*), or lemongrass oil (*45*).

### Evaluations of Minimum Risk 25(b) Exempt Active Ingredients against Ticks of Medical Concern in Europe

Studies on the castor bean tick (*Ix. ricinus*) have evaluated repellency of minimum risk 25(b) exempt active ingredients in laboratory assays (*47**–**49*). One noteworthy study (*47*) compared multiple minimum risk 25(b) exempt active ingredients, demonstrating sustained repellency against nymphal ticks up to 8 hours after application of 10% solutions of citronella oil (83% repellency by the eight-hour time point), clove oil (78%), and geraniol oil (67%). These compounds had similar or better repellency than a 10% DEET solution (71% repellency by 8 hours). 

In contrast, peppermint oil showed moderate repellency (50%) up to 4 hours but only minimal repellency after 6 hours (10%), and geranium oil had no repellency four hours after application.

## Conclusions

The review of published literature yielded more information for the effectiveness of minimum risk 25(b) exempt products intended to kill host-seeking ticks compared with tick repellent products. Considerable variability has been documented among marketed minimum risk 25(b) exempt products to kill host-seeking blacklegged ticks, with effectiveness similar to chemical products for some minimum risk products but minimal effect on ticks for other products. Moreover, different products based on the same active ingredients (e.g., rosemary and peppermint oils) can have highly variable tick killing efficacy, underscoring the difficulty in making recommendations about unregulated minimum risk products based solely on the active ingredients they contain. Evaluations of minimum risk 25(b) exempt tick repellents have typically focused on individual active ingredients rather than formulated commercial products, which often combine multiple active ingredients together with inert ingredients. In the near absence of studies on repellency of formulated products with similar and variable combinations of minimum risk active ingredients, it is not possible to make recommendations about unregulated minimum risk tick repellent products based solely on the active ingredients they contain. Consumers should be aware that effectiveness to kill and repel ticks can differ among unregulated minimum risk products, and independent sources of information on the effectiveness of specific products are most often lacking. There also is a need to better understand the reasons why members of the public choose to use EPA-registered versus minimum risk 25(b) exempt tick toxicant and repellent products, based on perceptions about effectiveness and safety for humans, pets, and the environment.
